# Management of pediatric obesity as a pathway towards kidney transplantation

**DOI:** 10.3389/fped.2024.1367520

**Published:** 2024-02-15

**Authors:** K. E. Altemose, C. Nailescu

**Affiliations:** Riley Hospital for Children, Division of Pediatric Nephrology, Indiana University School of Medicine, Indianapolis, IN, United States

**Keywords:** kidney, transplantation, children, obesity, adolescence

## Abstract

Obesity is an increasing problem in pediatrics, leading to cardiovascular, metabolic and psychosocial complications. Additionally, for patients with chronic kidney disease (CKD), obesity can lead to CKD progression towards end-stage renal disease (ESRD) needing renal-replacement therapy (RRT). It is well-established that the optimal type of RRT for children with ESRD is kidney transplantation, as it provides significantly better life expectancy and quality of life. Unfortunately, pediatric patients with CKD/ESRD and obesity face barriers getting to kidney transplantation and often remain on dialysis for a long time, which negatively impacts their life expectancy and quality of life. One barrier to kidney transplant is that Body Mass Index (BMI) is still considered by most transplant centers as the main criterion for obesity assessment, although more recent evidence suggests that BMI is not the best measure of adiposity. Clearcut evidence is lacking that obesity has a long-term negative impact upon the graft. Another barrier to transplant can be bias on the part of referring providers that can deter or delay referral to an obesity treatment program. Our article describes the barriers that pediatric obese patients with CKD and ESRD face in their way towards kidney transplantation. In addition, our article encourages pediatric nephrologists to early refer their patients with CKD and ESRD who suffer from obesity to a specialized obesity treatment program and/or bariatric surgery. Our article also describes the treatment options for pediatric patients with CKD and ESRD who suffer from obesity in order to make them eligible for a kidney transplant.

## 1 Introduction

Obesity in the pediatric population is an increasing problem. Between 1975 and 2016, the global age-standardised obesity prevalence world-wide increased from 0.7% (95% CI: 0.4–1.2) to 5.6% (CI: 4.8–6.5) for girls and from 0.9% (95% CI: 0.5–1.3) to 7.8% (CI: 6.7–9.1) for boys. Since 2000, this worrisome trend almost plateaued around these high levels in many high-income countries, but unfortunately has continued to rise in low and middleincome countries (1). It is estimated that by 2030, there will be 254 million children and adolescents with obesity across the globe (1). Specifically in the United States, as of 2017, the incidence of obesity among adolescents age 12–19 years was 21.2% (2).

According to the American Academy of Pediatrics (AAP), the definition of obesity is based on Body Mass Index (BMI) as follows: being overweight is charactetized by having a BMI between 85th percentile to less than the 95th percentile for age, or 25–29.9 kg/m^2^; Class 1 obesity is characterized by having a BMI between 95th percentile to less than 120% of the 95th percentile for age, or 30–34.9 kg/m^2^; Class 2 Obesity is characterized by having a BMI between 120% to less than 140% of the 95th percentile for age or BMI ≥ 35 to <40 kg/m^2^; Class 3 Obesity is characterized by having a BMI equal to 140% of the 95th percentile for age or over or BMI ≥ 40 kg/m^2^ (3).

The prevalence of obesity may be even higher in the pediatric chronic kidney disease (CKD) and end stage renal disease (ESRD) population, with studies citing prevalence of 12%–15% in the pediatric CKD and ESRD populations (4, 5). In addition to potential for developing adverse cardiovascular effects, metabolic syndrome, worsening of proteinuria and progression of CKD, pediatric patients with CKD/ESRD and obesity also unfortunately face barriers getting to kidney transplantation (6).

## 2 Selection for kidney transplant process for pediatric patients with obesity

### 2.1 The current selection process

The kidney transplant recipient selection process is rigorous and many criteria must be met for either approval for a living-donor transplant or activation on the United Network for Organ Sharing (UNOS) waiting list (7). One of the criteria for successful activation is generally a BMI within a certain range (4). The BMI cut-off criteria varies between transplant centers, but a survey by the AST (American Society of Transplantation) noted that 66 out of 67 surveyed centers used BMI in selection criteria, and the range of 35–45 kg/m^2^ as the upper limit for acceptance was used (8).

### 2.2 The rationale for the current selection process

Practice guidelines from the American Society of Transplantation pointed to increased risk for short-term and long-term complications as the reasoning behind BMI-based selection of transplant recipients (9). As far as short-term complications of obesity post-transplant, cardiovascular instability, delayed-graft function, impaired wound healing and increased risk for infections and surgical complications have been mentioned. As far as long-term complications, cardiovascular disease and possibly shorter graft survival have been suggested (9).

Short-term post-transplantation in patients with obesity, issues that have been cited as reasons for limiting transplantation in obese patients include that patients with obesity may become unstable at time of transplant. For example, approximately 60% of nondiabetic obese patients experience hyperglycemia in the immediate post-transplant phase (10). Obesity has been associated with delayed graft function – possibly related to longer time in the operating room (11). In addition, patients with obesity may have difficulty with wound healing and may develop infections and surgical complications due to larger incisions and also possible comorbid diabetes (12).

Regarding long-term complications post-transplantation in children with obesity, development of hypertension with associated increased odds of left ventricular hypertrophy and worse longitudinal strain have been reported (13). Obesity can lead to a reduction in glomerular filtration rate and proteinuria, which can contribute to graft failure over time (14). Additional risk factors that will expedite a patient's pathway to progressive CKD include comorbid complications that are typically associated with obesity, including hypertension, insulin resistance, hyperlipidemia and atherosclerosis (14). Obesity in pediatrics might be an independent risk factor for kidney transplant rejection and graft failure, as a negative correlation has been shown to exist between mean BMI and years of kidney graft survival (15). However, there is a lack of prospective study data on outcomes including graft loss or death after transplant in the obese adult transplant population, and even less data in the pediatric population (16). Therefore, using BMI cut-offs to restrict access to pediatric kidney transplantation may not be valid using the argument of potentially worsened graft function in patients suffering from obesity.

### 2.3 Problems with the current selection process

BMI as a criterion for selection may be more of a hindrance than a help. BMI is considered a controversial measure of adiposity, as it does not take muscle mass into consideration. It has been suggested that waist circumference is likely a better measure of abdominal adiposity (16). Waist circumference is known to be associated with increased cardiovascular disease risk (16). However, it has been noted that most patients with BMI over 35 kg/m^2^ will also have an abnormal waist circumference and thus an increased risk of cardiovascular disease (16). In addition, BMI does not take into account the inherent differences in adiposity between males and females (higher adiposity in females vs. higher muscle mass in males) (11). Currently there is no consensus among national organizations on definition of obesity using percentage of body fat, although there are multiple ways to measure body fat including bioelectrical impedance analysis (BIA), dual-energy x-ray absorptiometry (DXA) and hydrostatic weighing (9, 17).

BIA can measure body composition by applying a small alternating electrical current to the body and measuring electrical resistance (18). BIA devices from different manufacturers utilize mathematical models involving height, weight, age and sex for estimating the percentage of body fat (18). Several studies have shown BIA to be relatively accurate for estimating body composition in different adult populations, including healthy adults, malnourished ones, as well as those who suffer from obesity (19, 20). Emerging data exist in children, where BIA accurately estimated body fat percentage while also having the practical advantage of being done as a point of care test (21). DXA provides measures of body fat mass, lean mass, bone mineral content and body fat percentage and it has been shown to be relatively accurate, including in children (22). Unfortunately, DXA is not available as a point of care test and it involves radiation exposure, thus limiting its broad application in clinical practice (23). HW, also known as underwater weighing, hydrostatic body composition analysis or hydrodensitometry, basically measures body density using Archimedes' principle (24). Many equations have been published that reliably estimate body fat percentage from body density, both in general and special populations, however data in children is very limited (25). In addition, the equipment used is voluminous and not widely available, making its use very impractical (24). By accurately estimating the percentage of body fat, one can hope to better select transplant candidates, as some patients who were classified as obese based on BMI might have simply had larger bone structures and muscle mass, thus exaggerating the BMI and the degree of obesity. Unfortunately, while BMI may not be the ideal indicator of obesity, there is currently no other agreed-upon manner to assess it for transplant listing purposes.

A 2011 study showed that in hemodialysis patients, pre-transplant obesity was not associated with an increased risk of post-transplant graft failure or mortality, and instead a higher pre-transplant muscle mass was associated with better graft survival and decreased mortality (26). In theory, this may be due to improved nutrition and overall physical health in patients with a higher muscle mass than their counterparts with lower muscle mass (26).

### 2.4 Bias against patients suffering from obesity

Pediatric patients with obesity (even without CKD/ESRD) are at risk for bullying and stigmatization by their peers. Starting from an early age, children show weight-based stigma towards their peers suffering from obesity, viewing them as “ugly” and “lazy” (27). The stigmatization, however, does not stop at the peer level, as stigmatizing attitudes toward patients with obesity are expressed by medical professionals across diverse specialties (28).

A 2008 study looked at over 120,000 wait-listed patients in the United States and found that likelihood of being transplanted was inversely related to degree of obesity (thus more obese patients were less likely to receive a kidney transplant). The authors suggested that obese patients are considered less profitable than non-obese patients given risk for perioperative complications, and the fact that transplant centers may be penalized for poor outcomes if they occur in obese patients (29).

A study in 2014 found that for females, being classified as obese led to lower access to kidney transplantation compared to obese males and that time to transplantation after wait-listing was longer for obese women compared to obese men. This disparity was actually noted even starting with being overweight category. The authors pointed out that BMI is a potentially modifiable factor that can lead to sex-based inequity in access to transplantation (30).

In addition, there is also implicit bias on the part of physicians and even families that can deter or delay referral of pediatric patients to an obesity treatment program and/or bariatric surgery (31).

## 3 Non-pharmacological therapy

The American Board of Obesity Medicine (ABOM) pediatric obesity algorithm guidelines recommend that providers start management of obesity with intensive lifestyle therapy (including dietary counseling, physical exercise and shared decision making with the patient and family) and adjustment of any weight-promoting medications first (32).

Registered dieticians (RDs) play a critical role in obesity management, and weight management programs with RDs are more successful than those without them (33). Obesity can be addressed by addressing food insecurity. The prevalence of obesity is higher in adolescents from food-insecure households (34). Among healthy low-income children, monthly change in BMI is higher in food-insecure children (and thus they have a less optimal weight trajectory than food-secure children) (35). Food insecurity is known to be associated with increased hypertension, diabetes, heart disease, heart failure, stroke and CKD in adult patients (36). The associations between food insecurity and cardiometabolic outcomes have not been studied extensively in children.

Physical activity plays a very important role in prevention, as well as treatment of obesity (37). Physical activity should be adapted according to age and comorbidities (38). For example, the use of a pedometer has been associated with increased physical activity in adult patients, as well as a decrease in BMI. Having a step goal, such as 10,000 steps per day, has been associated with increased physical activity (39). In the pediatric population, having a pedometer with realistic goals like taking 500 steps/day above baseline has been found to significantly reduce BMI and to increase patients' subjective health and quality of life (40). It is also advisable to minimize screen time and promote other positive psychosocial influences, such as family meals, mindfulness during eating, stress management and emotional well-being, in order to prevent the onset of obesity (41).

The provider may consider advanced therapies such as pharmacotherapy or surgery in addition to intensive lifestyle therapy if needed (32).

## 4 Medical therapy

The ABOM recommends that a provider may start appropriate anti-obesity medication after the following are completed: careful history taking, physical exam, review of systems, review of medications which may prevent weight loss or promote weight gain, screening for obesity-related comorbidities, screening for genetic causes of obesity and screening for syndromic obesity (32).

Currently, there are only a few medications which are United States Food and Drug Administration (FDA)-approved for treatment of non-genetic causes of obesity in pediatric patients: Orlistat (for children 12 years and older) and Phentermine (for children 16 years and older) (42). All other obesity medications available in the United States are only FDA-approved for use in patients 18 years old and over, so use of these medications is considered off-label (42). Furthermore, randomized control trials supporting the safety and efficacy of these off-label medications in pediatric patients are limited (42). Therefore, caution is advised with prescription of off-label medications for pediatric use, and it is encouraged that an experienced pediatric obesity medicine provider and multidisciplinary team be involved in the care of pediatric patients suffering from obesity (42).

A 2021 meta-analysis examined the effects of glucagon-like peptide 1 (GLP-1) agonists in children less than 18 years old with obesity and/or type 2 diabetes (43). The authors found that GLP-1 agonists reduced body weight more in children with obesity than children with type 2 diabetes, and that the effect sizes were similar to adult studies (43). They also found mild side effects including gastrointestinal symptoms and mild hypoglycemia, but no serious adverse events (43). Currently, GLP-1 agonists are only approved by FDA for children with type 2 diabetes who are 10 years old and older (43). However, the authors suggested that GLP-1 agonists can be considered for treatment of pediatric obesity as they are relatively effective and safe (43).

Selective sodium-glucose cotransporter 2 (SGLT2) inhibitors, approved for the treatment of type 2 diabetes in adults, function via an insulin-independent mechanism for improving blood glucose levels (44). SGLT2 inhibitors enhance glucose excretion in the urine by almost 50% by inhibiting the reabsorption of filtered glucose in the proximal tubules (44). In addition, SGLT2 inhibitors have been also found to promote weight loss (45). A meta-analysis of adult patients with type 2 diabetes showed that SGLT2 inhibitors use was associated with a decrease in body weight of 2.01 kg (95% CI: −2.18 to −1.83 kg) compared to baseline (45). The mechanism is not well-understood, however, seems to be beyond what can be simply explained by glycosuria and its resulting energy deficit and also beyond water excretion via osmotic diuresis (44). However, SGLT2 inhibitors are not approved specifically for weight loss in the absence of diabetes in any patient population. SGLT2 inhibitors use is not approved for patients age less than 18. However, one SGLT2 inhibitor in particular, Canagliflozin, is currently in trial in children age 10–17 with type 2 diabetes (NCT03170518).

In addition to medical pharmacologic treatments of obesity, psychologic/psychiatric therapies should be considered both pre- and post-transplant, as obesity is associated with an increased risk of depression (46). This is particularly important during adolescence and the effect is amplified in female patients vs. males (47). Depression leads to decreased medication adherence and increased risk of graft rejection and failure (48). Depression in turn is also associated with an increased risk of obesity, thus creating a vicious cycle (49). The development of depression and psychological comorbidities may be exacerbated by the fact that obese patients are often bullied in childhood and under stigmatization by their peers (27). In conclusion, identifying psychologic problems that can be contributing to obesity, such as depression, may ultimately allow patients to receive treatment and to set them up for success in both the pre- and post-transplant periods.

Regardless of what type of therapy is initiated, ABOM stresses that obesity is a chronic disease and patients should be counseled regarding the need for lifetime treatment. The focus of providers should be managing instead of simply trying to “cure” obesity (32).

## 5 Surgical therapy

For patients suffering from obesity who have not succeeded with dietary and lifestyle interventions and medical management, bariatric surgery can be considered. The two procedures that are most commonly used are laparoscopic sleeve gastrectomy and laparoscopic Roux-en-Y gastric bypass. The most optimal timing (pre- or post-transplantation) is still controversial, but bariatric surgeons tend to prefer pre-transplant rather than post-transplant surgery (50).

The AAP Policy Statement from 2019 stated that children meeting BMI cutoff of greater than 40 kg/m^2^ or 140% of the 95th percentile are unlikely to benefit from lifestyle modification or pharmacotherapy alone, and therefore should be considered for bariatric surgery (51). A similar recommendation was made for children with a BMI greater than 35 kg/m^2^ or 120% of the 95th percentile with an obesity-related comorbidity. Contraindications to bariatric surgery include substance abuse, pregnancy, untreated mental illness or an untreated underlying medical cause of obesity. Patients with developmental delay and autism spectrum disorder can be considered for bariatric surgery (31).

Pre-transplant sleeve gastrectomy has been shown to increase access to the transplant waitlist (52). Patients can achieve approximately 60%–80% excess body weight loss within 18–24 months (50). Bariatric surgery may improve renal function in patients with CKD who are pre-dialysis, and has also been shown to decrease post-transplant graft failure and mortality, as well as decrease need for antihypertensive medications and insulin in patients suffering from diabetes (17). However, it is important that in addition to the surgical treatment, patients receive care and support of a multidisciplinary team including a psychologist and/or psychiatrist, nutritionist, bariatric surgeon, and nephrologist (17).

## 6 Conclusions

The first step towards optimizing the selection process for kidney transplantation for pediatric patients suffering from obesity should consist of establishing better criteria. Relying simply on BMI no longer seems to be the most accurate method to assess excess adiposity and therefore we might need to include other assessment tools, such as bioelectrical impedance analysis, dual-energy x-ray absorptiometry or hydrostatic weighing. An individualized approach that would include the patient and the immediate family should be applied. Lifestyle modification, medical therapy and if necessary, psychological/psychiatric and surgical therapy, should be used on a case-by-case basis. We recommend, based on review of the evidence, that obesity be assessed and addressed pre-transplant by a multidisciplinary team including but not limited to a psychologist and/or psychiatrist, nutritionist, nephrologist and bariatric surgeon. This approach can position pediatric kidney transplant candidates suffering from obesity to have the best access to transplantation, as well as the best prognosis after transplant.
